# Identifying metabolic bottlenecks for micafungin precursor production via untargeted regulatory perturbation

**DOI:** 10.1186/s13068-026-02737-7

**Published:** 2026-01-14

**Authors:** Ping Men, Li Xie, Jiachen Wang, Yu Zhou, Xiaoxi Zhang, Yanping Li, Xuenian Huang, Xuefeng Lu

**Affiliations:** 1 Shandong Provincial Key Laboratory of Synthetic Biology, Qingdao Institute of Bioenergy and Bioprocess Technology, Chinese Academy of Sciences, Qingdao, 266101 China; 2 Shandong Energy Institute, Qingdao, 266101 China; 3 Qingdao New Energy Shandong Laboratory, Qingdao, 266101 China; 4 University of Chinese Academy of Sciences, Beijing, 100049 China; 5 College of Life Sciences, Qingdao Agricultural University, Qingdao, 266109 China; 6 Institute for Smart Materials & Engineering, University of Jinan, Jinan, 250022 China; 7 State Key Laboratory of Food Science and Resources, Nanchang University, Nanchang, 330047 China; 8 Marine Biology and Biotechnology Laboratory, Qingdao National Laboratory for Marine Science and Technology, Qingdao, 266237 China

**Keywords:** Micafungin, *Coleophoma empetri*, Untargeted regulatory perturbation strategy, Transcriptome analysis, High-titer mechanism

## Abstract

**Background:**

Micafungin, a clinically important echinocandin antifungal agent, is derived from the nonribosomal cyclic hexapeptide FR901379 produced by the filamentous fungus *Coleophoma empetri*. However, low fermentation efficiency remains a major constraint in its industrial production.

**Results:**

In this study, we implemented an untargeted regulatory perturbation strategy to systematically identify metabolic bottlenecks affecting FR901379 biosynthesis. A mutant library was constructed by rationally engineering the key untargeted regulatory genes involved in histone modification and global regulation. The untargeted perturbation led to diverse phenotypes in both growth and secondary metabolism, ranging from enhancement (by up to 170%) to complete abolition of FR901379 production. Transcriptome profiling of high-producing strains revealed coordinated upregulation of genes in the acetyl-CoA, palmitic acid, and 3′-phosphoadenosine-5′-phosphosulfate biosynthetic pathways. Exogenous supplementation of palm oil further enhanced FR901379 titer by 87.6%, confirming the critical role of precursor supply.

**Conclusions:**

This work elucidates the metabolic network governing FR901379 biosynthesis and provides key candidates for further metabolic engineering. It also demonstrates that untargeted regulatory perturbation strategy is an effective approach for deciphering the mechanisms behind specific phenotypic traits in industrial filamentous fungi.

**Supplementary Information:**

The online version contains supplementary material available at 10.1186/s13068-026-02737-7.

## Background

Micafungin is an important echinocandin-type antifungal agent used for the clinical treatment of invasive fungal infections (IFIs) [1]. It is derived from the cyclic hexapeptide FR901379 produced by the filamentous fungus *Coleophoma empetri* [2, 3]. The low fermentation efficiency of FR901379 remains a major bottleneck in reducing the production cost of micafungin.

Recent studies have shown that the biosynthesis of FR901379 requires two discrete gene clusters, a hexapeptide cluster and a sulfonation cluster. The expression of these two gene clusters was governed by the pathway-specific transcription factor McfJ in a coordinated way [4]. Based on this insight, a highly efficient FR901379-producing strain was constructed by overexpressing *mcfJ* along with key rate-limiting enzymes [5]. As a result, the titer of FR901379 increased from 0.3 g/L to 2.03 g/L in the shake flask [5]. Overexpression of pathway-specific transcription factors offers a versatile strategy for enhancing microbial biosynthesis. This approach has been successfully applied in rational engineering of other industrial echinocandin-producing strains, including pneumocandin B_0_ and echinocandin B [6, 7]. However, further increasing the copy number of *mcfJ* did not lead to additional improvements in FR901379 production. These results suggest that metabolic bottlenecks beyond the core biosynthetic pathway may limit the increase in production. The key restriction is that the chassis cell and the biosynthetic pathway are not compatible.

The adequate supply of key precursors, such as amino acids and fatty acids, is crucial for the efficient synthesis of echinocandins. For instance, the titer of echinocandin B was significantly increased upon the addition of methyl oleate [8, 9], indicating that limited precursor availability is a major bottleneck in achieving high titers. The biosynthesis of these precursors is intrinsically complex, involving multiple interconnected metabolic processes including glycolysis, tricarboxylic acid cycle (TCA), pentose phosphate pathway (PPP), amino acid metabolism, and fatty acid metabolism [10, 11]. These metabolic pathways involve the concerted regulation of multiple genes [12–15]. Mutation breeding has long been an important method for screening high-yielding industrial strains, as it can improve production performance, fermentation robustness, and mycelial morphology [16–19]. However, since mutagenesis randomly introduces numerous irregular mutations, it is often challenging to elucidate the mechanisms underlying the high productivity of mutant strains. This ambiguity hinders further rational metabolic engineering. Therefore, it is crucial to provide an effective strategy that can rapidly and accurately identify key metabolic processes or target genes within the metabolic network to facilitate the rational design of high-yielding strains.

In filamentous fungi, the functional expression of genes involves a multi-step process under complex regulatory control [20–23]. Among these mechanisms, histone modifications and global regulators play critical roles in activating cryptic secondary metabolism and enhancing the production of valuable compounds [23–25]. Histone methylation and acetylation, which primarily occur on lysine residues, regulate secondary metabolism through chromatin-mediated processes [24, 26, 27]. For example, disruption of CclA, an ortholog of histone H3 lysine 4 methyltransferase, resulted in overproduction of gliotoxin in *Aspergillus fumigatus* [28]. In addition, inactivation of a histone H3 deacetylase HdaA led to pleiotropic activation and overexpression of more than 75% of the biosynthetic genes in *Calcarisporium arbuscula* [24]. The conserved COP9 signalosome (CSN) plays a central role in regulating post-translational modifications, such as protein ubiquitination, de-ubiquitination, and phosphorylation [25, 29]. Deletion of the CSN subunit-encoding gene *PfcsnE* resulted in the increased production of metabolite chloroisosulochrin in *Pestalotiopsis fici* [25]. In addition, secondary metabolism is subject to global epigenetic control at a higher hierarchical level. LaeA, a nuclear protein widely conserved in numerous filamentous fungi, has been identified as a global regulator of secondary metabolism [30]. It is proposed that LaeA modulates chromatin accessibility to transcription factors at the regions of secondary metabolite gene clusters, thereby influencing their expression [21, 31–34]. LaeA, together with VeA and VelB, forms a heterotrimeric complex present in most filamentous fungi. It coordinately regulates the biosynthesis of diverse secondary metabolism, such as echinocandin B, sterigmatocystin, penicillin, aflatoxin, lovastatin, gliotoxin, and many others [30, 35]. Given that both FR901379 and echinocandin B share a cyclic hexapeptide structure and possess highly similar biosynthetic pathways, we reasoned that their regulatory mechanisms might also be conserved. Although the mechanisms underlying these regulatory processes are partially deciphered, their precise gene targets remain largely elusive. Thus, histone modifications and global regulators can be categorized as a form of “untargeted regulation”. This feature provides a rational basis for metabolic engineering strategies employing untargeted perturbations to increase secondary metabolite production.

**Fig. 1:**
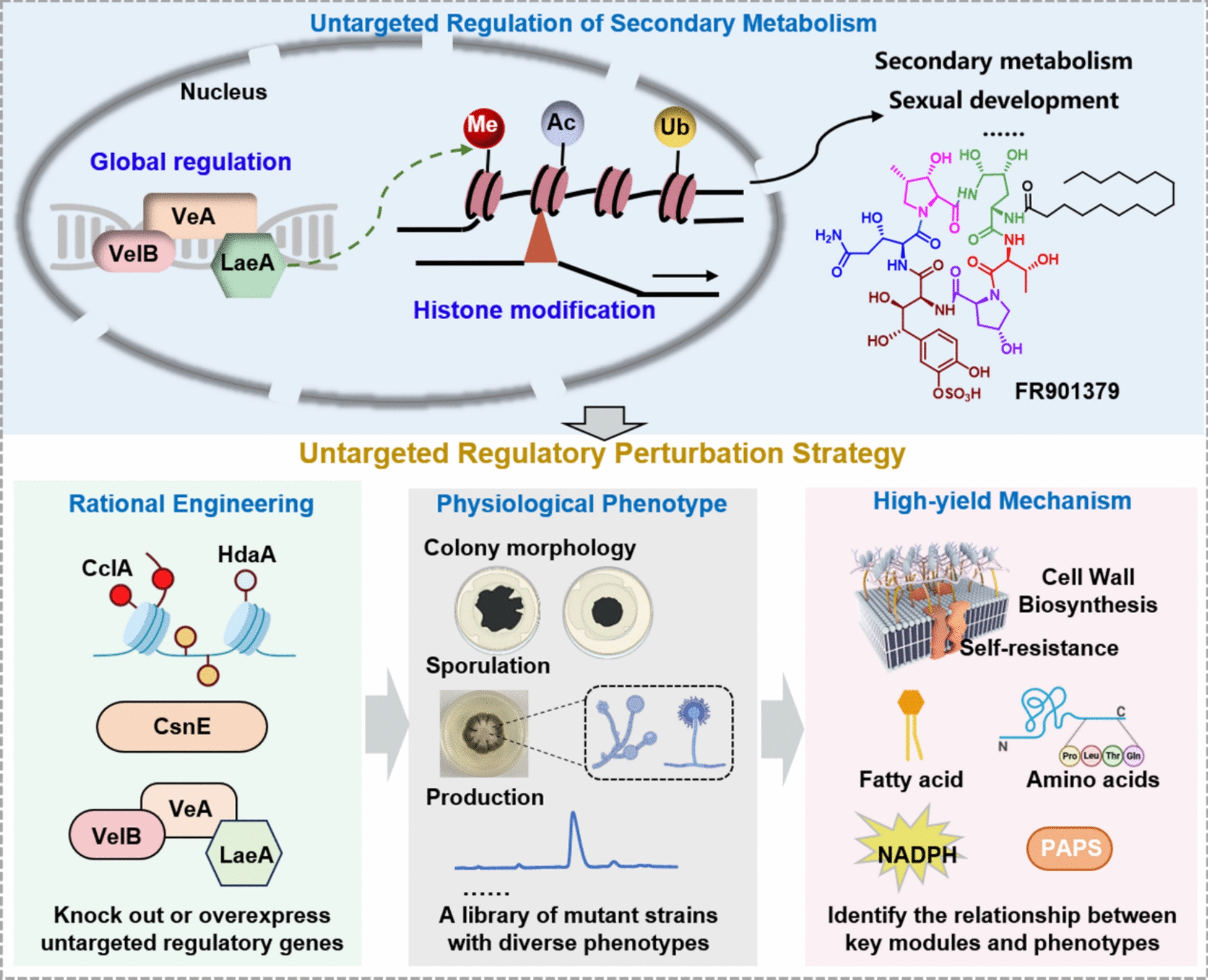
Schematic diagram of untargeted regulation of secondary metabolism and URPS

In this study, we developed an untargeted regulatory perturbation strategy (URPS) for rapid identifying metabolic and regulatory modules associated with physiological phenotypes in *C. empetri* (Fig. 1). Specifically, we constructed a library of functional strains exhibiting diverse physiological phenotypes by knocking out or overexpressing key untargeted regulatory genes. The function of selected untargeted regulatory factors has been validated across diverse filamentous fungi, histone modifications (*CecsnE*, *CecclA*, and *CehdaA*) and global regulators (*CelaeA*, *CeveA*, and *CevelB*) were prioritized in this study. Subsequently, transcriptome analysis was performed to identify the relationship between key modules and phenotypes and elucidate the high-titer mechanism of FR901379. This study introduces a novel untargeted perturbation engineering strategy integrated with transcriptome analysis, which facilitates the elucidation of high-titer mechanisms and supports the rational design of high-yielding strains.

## Materials and methods

### Strains and culture conditions

The wild-type strain *C. empetri* MEFC09 and its derived strains are listed in Table 1. These strains were cultivated at 25 °C on potato dextrose agar (PDA) plates.

**Table 1:** Strains used in this study

Strains	Genotype	References
MEFC09	Parent strain, *Coleophoma empetri*	CGMCC^a^ 21058
MEFC10	Δ*ku80*::*G418*	[36]
Δ*CecclA*	Δ*ku80*::*G418*, Δ*CecclA*::*hph*	This study
Δ*CehdaA*	Δ*ku80*::*G418*, Δ*CehdaA*::*hph*	This study
Δ*CecsnE*	Δ*ku80*::*G418*, Δ*CecsnE*::*hph*	This study
Δ*CelaeA*	Δ*ku80*::*G418*, Δ*CelaeA*::*hph*	This study
Δ*CeveA*	Δ*ku80*::*G418*, Δ*CeveA*::*hph*	This study
Δ*CevelB*	Δ*ku80*::*G418*, Δ*CevelB*::*hph*	This study
OE*CecclA*	PgpdAt-CecclA-Tpgk, *hph*	This study
OE*CehdaA*	PgpdAt-CehdaA-Tpgk, *hph*	This study
OE*CelaeA*	PgpdAt-CelaeA-Tpgk, *hph*	This study
OE*CeveA*	PgpdAt-CeveA-Tpgk, *hph*	This study
OE*FAS1*	Δ*ku80*::*G418*, PgpdAt-FAS1-Tpgk, *hph*	This study
OE*ACC1*	Δ*ku80*::*G418*, PgpdAt-ACC1-Tpgk, *hph*	This study

^a^China General Microbiological Culture Collection Center

### Construction of knockout strains

All primers and plasmids used in this study are listed in Table S1 and Table S2, respectively. The strategy for gene knockout was carried out in MEFC10 as shown in Fig. S1. For gene knockout, about 1.2 kb fragments of upstream and downstream of the target genes were amplified from the genomic DNA of MEFC09. The marker gene *hph* was amplified by using primers hph-F/hph-R with the plasmid pXH2-1 as a template [37]. Then fragments of upstream and downstream were fused with marker *hph* by fusion PCR. The targeting fragments were amplified from fusion PCR using nest primers and purified by Cycle-Pure Kit (Omega Bio-Tek, Guangzhou, China).

The knockout strains were generated through PEG/CaCl_2_-mediated protoplast transformation following established protocols [36]. Hygromycin B resistant colonies were selected after incubating on PDAS-H plates (PDA with 0.8 M *D*-sorbitol and 100 µg/mL hygromycin B) for 5–7 days. The knockout strains were verified by PCR using the outside primers (Fig. S3).

### Construction of overexpressing strains

For gene overexpression, the target genes were amplified from the genomic DNA of MEFC09 and inserted into the *Xba* I site of the vector pU19-ZX [5] by using a One-Step Cloning Kit (Vazyme, Nanjing, China) to achieve the recombinant plasmids pU-CecclA, pU-CehdaA, pU-CelaeA, pU-CeveA, pU-FAS1, and pU-ACC1 (Fig. S2). All plasmids were confirmed by sequencing analysis. The expression cassettes were amplified from the corresponding plasmids using primers PgpdAt-F/hph-R and introduced into the MEFC09. These overexpressing strains were verified by PCR using the primers as shown in Fig. S4.

To validate the functions of key genes in the palmitic acid biosynthesis pathway, we overexpressed these genes at the neutral locus *cemelR* via homologous recombination. The upstream and downstream homologous arms of *cemelR* were inserted into the empty vector pU19 to achieve the plasmid pU-cemelR. And then fragment PgpdAt-FAS1-Tpgk was inserted into the *Xba* I site of the vector pU19-cemelR to achieve the recombinant plasmid pU-cemelR-FAS1. Plasmid pU-cemelR-ACC1 was obtained by the same method. The expression cassettes were amplified from the corresponding plasmids using primers UcemelR-CS-F/DcemelR-CS-R and introduced into the MEFC10. These overexpressing strains were verified by PCR using the primers PgpdAt-F/Tpgk-R.

### Hyphal growth and morphology observation

To measure the colony diameters and observe the morphology, the seven strains of MEFC10, ∆*CecclA,* ∆*CehdaA*, ∆*CecsnE*, ∆*CelaeA*, ∆*CeveA*, and ∆*CevelB* were grown on PDA plates at 25 °C for 11 days. Hyphal growth was measured at two-day intervals, and morphology was observed daily.

### Fermentation and metabolite profiling of the engineered strains

Approximately 2 × 2 cm^2^ mycelium were crushed incubated into 50 mL of MKS medium (15 g/L soluble starch, 10 g/L sucrose, 5 g/L cottonseed meal, 10 g/L peptone, 3 2 g/L CaCO_3_ and 1 g/L KH_2_PO_4_; pH 6.5) in 250 mL shake flasks for 40–44 h at 25 °C and 220 rpm. Then 5 mL of seed culture was inoculated into 50 mL MKF medium (10 g/L glucose, 160 g/L *D*-sorbitol, 30 g/L corn starch, 10 g/L peptone, 6 g/L (NH_4_)_2_SO_4_, 1 g/L KH_2_PO_4_, 0.3 g/L FeSO_4_·7H_2_O, 0.01 g/L ZnSO_4_·7H_2_O, 2 g/L and CaCO_3_; pH 6.5) for additional 8 days at 25 °C and 220 rpm. Afterwards, the mixture of 1 mL of fermentation broth and 3 mL methanol was shaken for 1 h at room temperature and centrifuged at 12,000 × *g* for 20 min before high performance liquid chromatography (HPLC) analysis. Three independent experiments were performed for each transformant.

The separation was performed on an Agilent 1260 system with a DAD detector equipped using an Agilent ZORBAX SB-C18 column (4.6 mm by 150 mm with a 5 µm particle size) at a flow rate of 1 mL/min. The linear gradients system for HPLC is 0–5 min 5%-40% A, 5–15 min 40%–60% A, 15–20 min 100% A, 20–25 min 5% A (A: acetonitrile with 0.05% trifluoroacetic acid, B: deionized water with 0.05% trifluoroacetic acid).

### RNA isolation and transcriptome analysis

Mycelium were collected from the MKF cultures at different time intervals (48 h and 96 h) and immediately frozen in liquid nitrogen. Mycelium samples were sent to Azenta (Suzhou, China) for transcriptome sequencing by Illumina Hiseq X Ten system. The corresponding expression levels were obtained by calculating fragments per kilobase per million reads (FPKM). The results were illustrated using TBtools based on the Euclidean distance calculation.

## Results

### Bioinformatic prediction of genes related to untargeted regulatory

To assess the impact of untargeted regulation on FR901379 biosynthesis, we systematically evaluated the roles of histone methyltransferase, histone deacetylase (HDAC), CSN, and the global regulators LaeA, VeA, and VelB. The encoding genes of *CecsnE*, *CecclA*, *CehdaA*, *CelaeA*, *CeveA*, and *CevelB* in MEFC09 were mined out through bioinformatic analysis according to the corresponding homologous genes identified in *Aspergillus nidulans* FGSC A4 [26, 34]. These candidate genes are highly similarity to those in *A. nidulans* FGSC A4 (Table 2), suggesting that they may serve as the regulator of secondary metabolism.

**Table 2:** Genes associated with untargeted regulation in MEFC09

	Histone modifications			Global regulators		
Gene name	*CecclA*	*CehdaA*	*CecsnE*	*CelaeA*	*CeveA*	*CevelB*
Identity	35%	48%	64%	44%	60%	44%
Similarity	49%	64%	77%	59%	73%	54%
Reference sequence in NCBI	AN9399.4	AN8042.4	AN2129.4	AN0807.4	AN1052.4	AN0363.4

### Regulation on morphology and FR901379 production

**Fig. 2:**
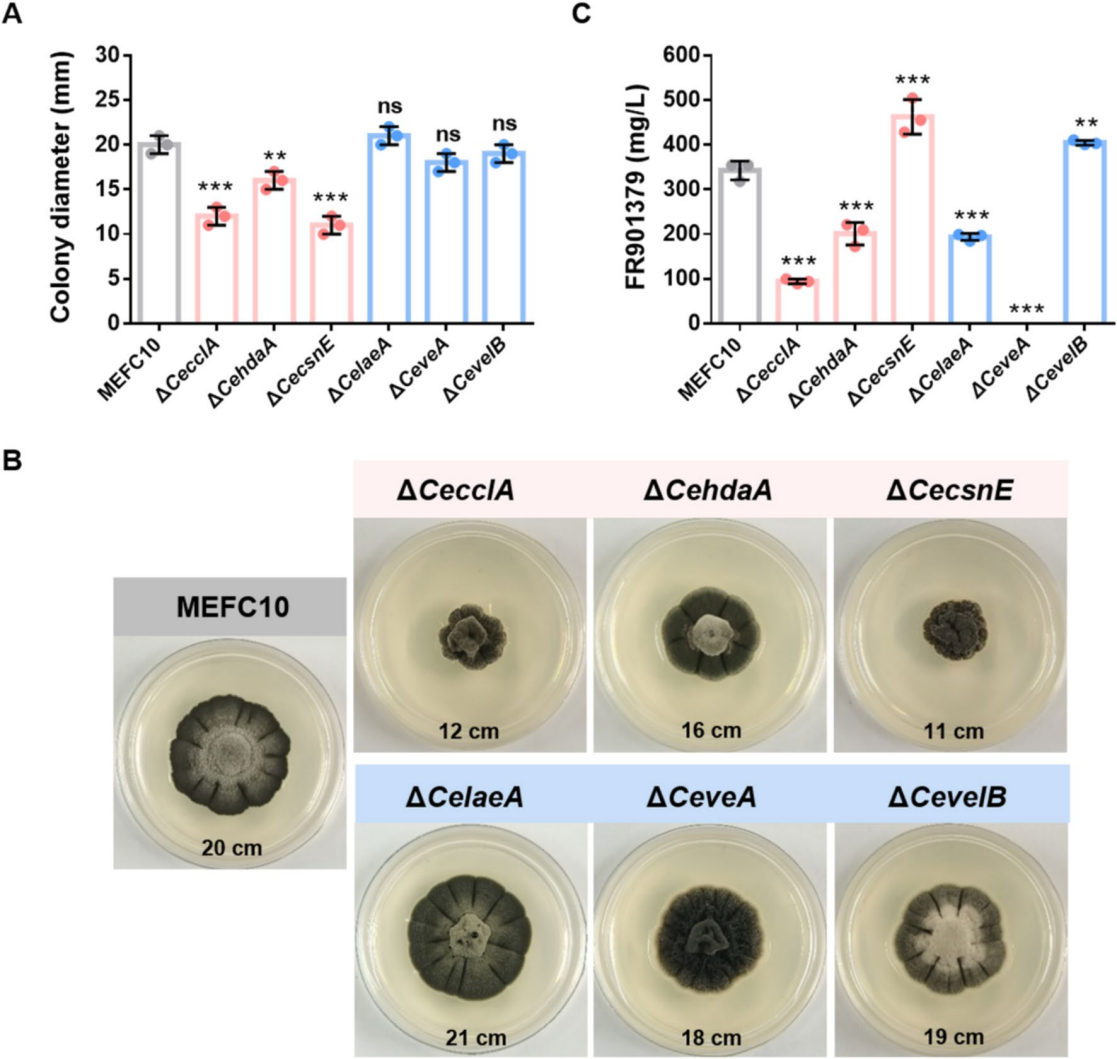
Colony morphology and FR901379 titers of MEFC10 and the deletion mutants. **A** The colony diameter of MEFC10 and mutant strains. **B** The morphology of MEFC10 and mutant strains on the PDA plates. **C** The FR901379 titers of MEFC10 and mutant strains. Data of (**A**, **C**) are mean ± SD from *n* = 3 biologically independent samples. Statistical analyses were performed using one-way ANOVA followed by Dunnett’s multiple comparison test. **P < 0.01, ***P < 0.001, ns: no significant difference

To investigate the functions of these untargeted regulatory genes in *C. empetri,* all genes listed in Table 2 was individually knocked out in the non-homologous end joining (NHEJ) deficient strain MEFC10. The growth rate, colony morphology and FR901379 production of MEFC10 are the same as those of the wild-type strain MEFC09 [36]. The mutant strains were evaluated for physiological phenotypes including growth, morphological development, and FR901379 production. After 11 days of cultivation on PDA plates, the colony diameters of the MEFC10, ∆*CecclA*, ∆*CehdaA*, and ∆*CecsnE* were 20 ± 1, 12 ± 1, 16 ± 1, and 11 ± 1 mm, respectively (Fig. 2A). Disruption of *CecclA*, *CehdaA*, and *CecsnE* resulted in reduced growth rates. In contrast, deletion of the global regulatory genes *CelaeA*, *CeveA*, or *CevelB* did not significantly affect radial rates (Fig. 2A).

All mutant strains displayed distinct morphological alterations compared to the parental strain. The ∆*CecclA* and ∆*CecsnE* mutants formed dense mycelia, although the ∆*CeveA* mutant exhibited a markedly fluffier mycelial morphology. The Δ*CehdaA* mutant showed reduced radial furrowing, whereas the Δ*CelaeA* mutant displayed more extensive and pronounced furrows (Fig. 2B). Notably, the ∆*CevelB* mutant exhibited a gray colony surface covered with a dense layer of conidia (Fig. 2B). These findings demonstrate that *CecclA*, *CehdaA*, and *CecsnE* are involved in regulating vegetative growth, although *CeveA*, *CelaeA*, and *CevelB* differentially influence colony morphology and asexual development, particularly conidiation and hyphal structure.

To evaluate the roles of these six genes in FR901379 biosynthesis, all mutant strains along with the MEFC10 control strain were subjected to for 8-days fermentation, followed by HPLC analysis to quantify FR901379 titers. Compared with MEFC10, the production of FR901379 in ∆*CecclA*, ∆*CehdaA*, and ∆*CelaeA* were reduced 72%, 41%, and 43%, respectively. In contrast, deletion of *CeveA* completely abolished FR901379 production. Notably, the Δ*CecsnE* mutant exhibited a 35% increase in FR901379 titer relative to MEFC10 (Fig. 2C). These results demonstrate that CeVeA is essential for FR901379 biosynthesis, CeCclA, CeHdaA, and CeLaeA serve as positive regulators, and that CeCsnE functions as a negative regulator.

### Overexpressing positive regulators to increase FR901379 production

**Fig. 3:**
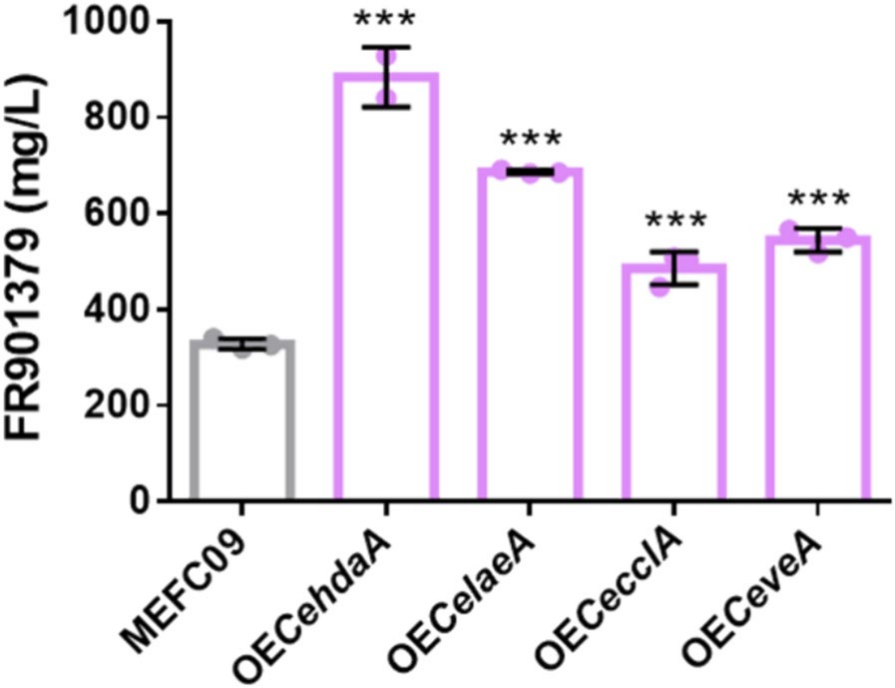
Titers of FR901379 in MEFC09 and overexpression strains OE*CehdaA*, OE*CelaeA*, OE*CeveA*, and OE*CecclA*. All data are mean ± SD from *n* = 2 or 3 biologically independent samples. Statistical analyses were performed using one-way ANOVA followed by Dunnett’s multiple comparison test, ***P < 0.001

Since knockout of *CecclA*, *CehdaA*, *CelaeA*, and *CeveA* significantly reduced FR901379 production, we hypothesized that overexpressing these genes would enhance its biosynthesis. To test this hypothesis, expression cassettes for *CecclA*, *CehdaA*, *CelaeA*, and *CeveA* were individually introduced into MEFC09, generating the overexpression strains OE*CecclA*, OE*CehdaA*, OE*CelaeA*, and OE*CeveA*, respectively (Fig. S4). Fermentation analysis revealed that the overexpression of all four genes significantly increased FR901379 production (Fig. 3). The most pronounced effect was observed in the OE*CehdaA* strain, which achieved a titer of 884.5 mg/L, a 170% increase over to the parental strain. The OE*CelaeA*, OE*CeveA*, and OE*CecclA* strains also showed substantial improvements, producing 687.1 mg/L, 544.8 mg/L, and 486.4 mg/L, corresponding to increases of 109%, 66%, and 48%, respectively.

These results confirm that *CehdaA*, *CelaeA*, *CecclA*, and *CeveA* act as positive regulators of FR901379 biosynthesis, with *CehdaA* overexpression yielding the most significant enhancement. It also highlights the potential of targeting these untargeted regulatory genes in metabolic engineering strategies for improved FR901379 production. However, the mechanisms by which these untargeted regulators affect the strain phenotype require further investigation. This also presents a valuable opportunity to identify critical metabolic modules associated with product synthesis.

### Effect on the transcription of the FR901379 biosynthetic gene cluster

**Fig. 4:**
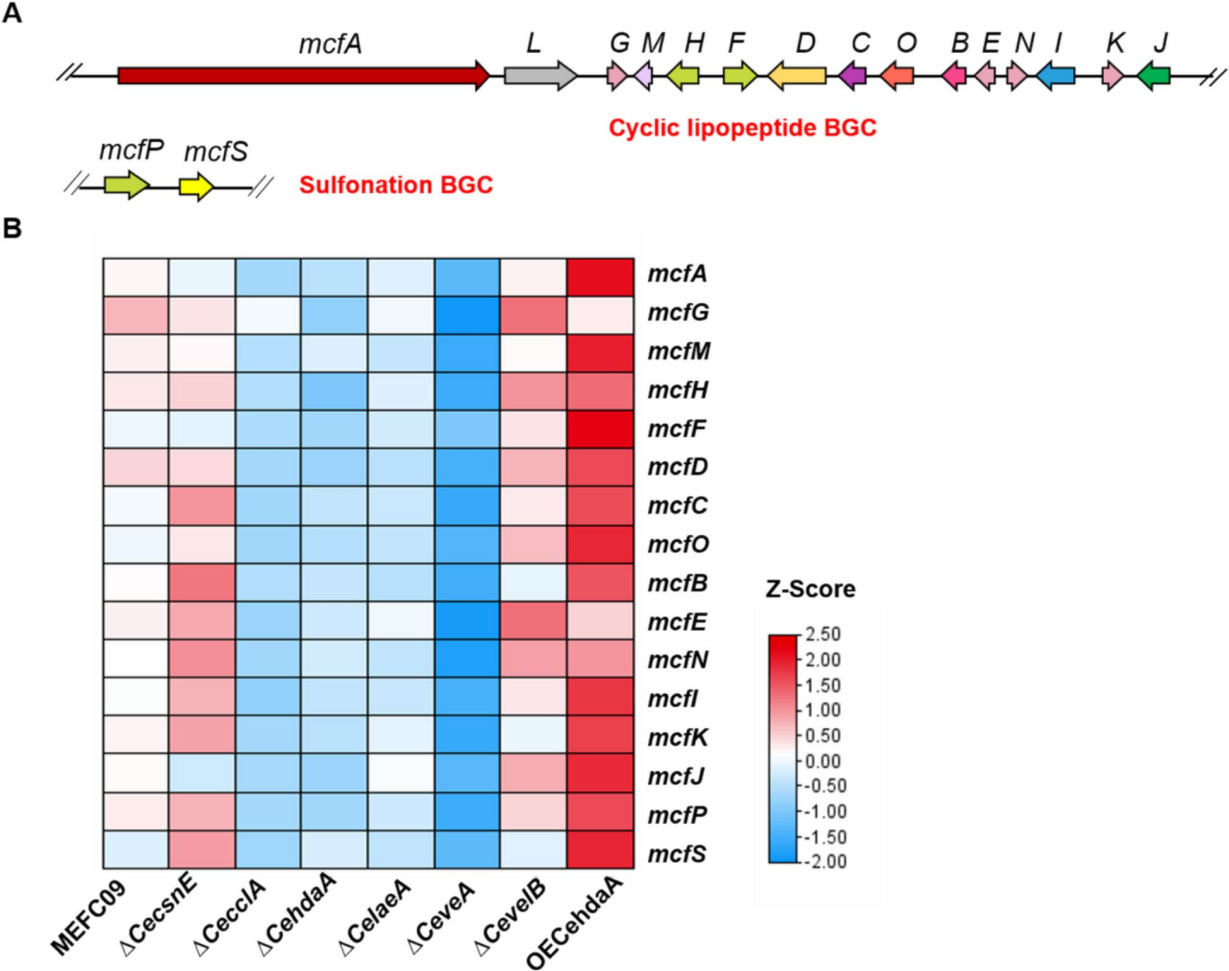
Transcriptome analysis of FR901379 biosynthetic genes. **A** The biosynthetic gene clusters of FR901379, BGC: biosynthetic gene cluster. **B** Heatmap of the *mcf* gene expression profile of MEFC09, ∆*CecsnE*, ∆*CecclA,* ∆*CehdaA*, ∆*CelaeA*, ∆*CeveA*, ∆*CevelB*, and OE*CehdaA*. Relative expression levels are shown as a color gradient from low (blue) to high (red)

The biosynthesis of FR901379 is mediated by sixteen genes distributed across two discrete clusters (Fig. 4A). To investigate how the transcription levels of these genes is influenced by untargeted regulators, RNA-seq analysis was performed on all knockout mutants and the OE*CehdaA* overexpression strain. Knockout of *CecclA*, *CehdaA*, and *CelaeA* led to significant downregulation of the FR901379 biosynthetic genes. In the ∆*CeveA* mutant, transcription of these genes was nearly completely abolished, aligning with the sharp decline in FR901379 production (Fig. 4B). Overexpression of the CeHdaA significantly upregulated the transcription of most *mcf* genes. These results demonstrate that the disruption of either CeCclA-mediated histone methylation, CeHdaA-mediated histone deacetylation, or the global regulators CeLaeA and CeVeA significantly inhibits the biosynthesis of FR901379. Conversely, upregulating histone deacetylation mediated by CeHdaA, which reduces histone acetylation, promotes FR901379 biosynthesis.

### Transcriptional analysis of chassis metabolic pathways

**Fig. 5:**
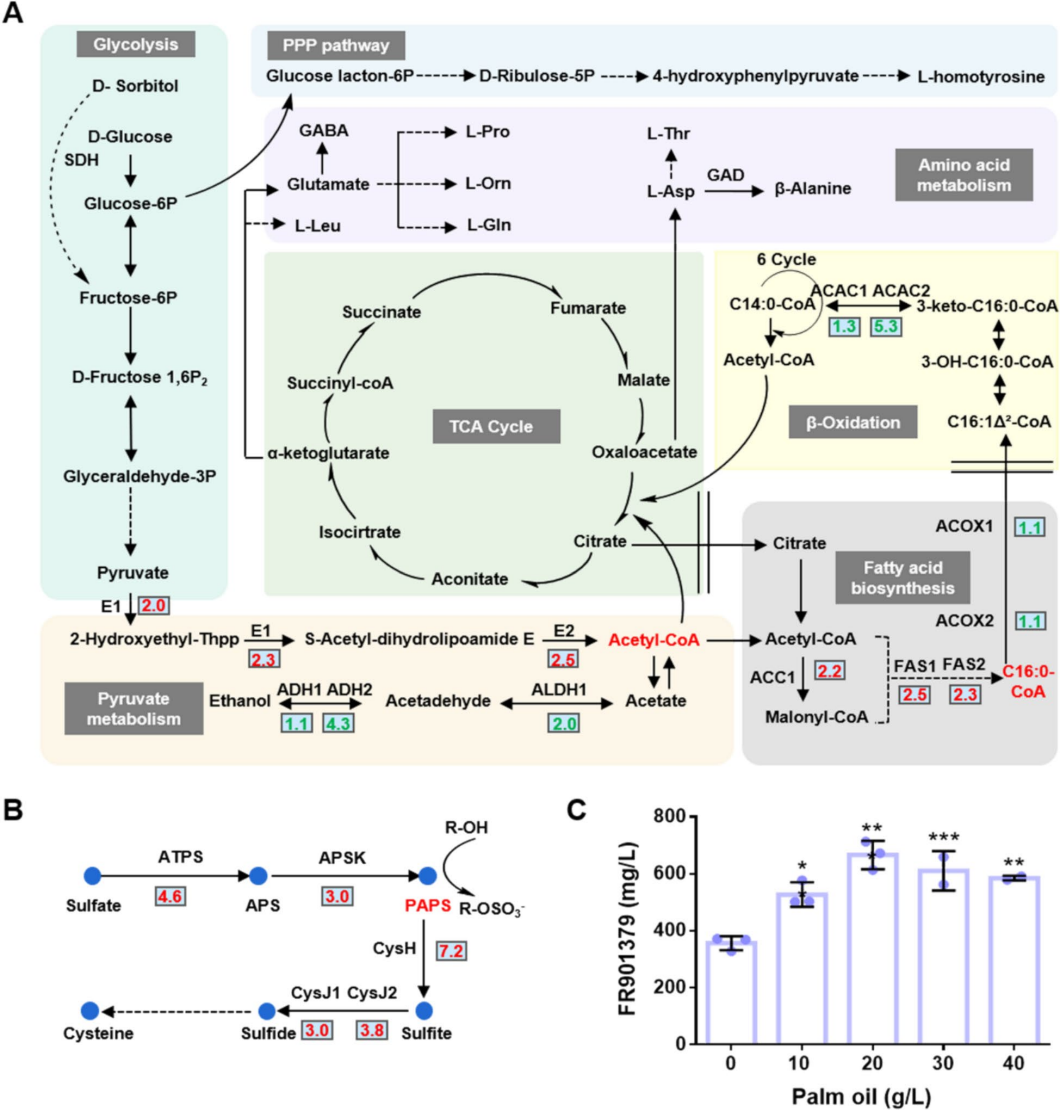
Transcriptome analysis of chassis metabolic pathways and production analysis of FR901379. **A** The expression levels of key genes in FR901379-related metabolic pathways in OE*CehdaA*. Red color indicates upregulation of gene expression levels; green color indicates downregulation; The numbers next to genes represent log2(fold change), with red and green indicating significant upregulation and downregulation, respectively; E1: pyruvate dehydrogenase E1 component; E2: pyruvate dehydrogenase E2 component; ACC1: acetyl-CoA carboxylase; FAS1/ FAS2: fatty acid synthase; ACOX1/ACOX2: acyl-CoA oxidase; ACAC1/ACAC2: acetyl-CoA acyltransferase; ALDH1/ALDH2: aldehyde dehydrogenase; ADH1/ADH2: alcohol dehydrogenase; GAD: glutamate decarboxylase; SDH: sorbitol dehydrogenase; GABA: 4-Aminobutanoic acid. **B** The upregulation levels of key genes in the PAPS synthesis pathway in the mutant strains ∆*CecsnE* and OE*CehdaA.* ATPS: ATP sulfurylase; APS: adenosine 5'-phosphosulfate, APSK: Adenylylsulfate kinase; CysH: phosphoadenosine phosphosulfate reductase, CysJ: sulfite reductase. **C** Effect of palm oil concentration on FR901379 production. All data are mean ± SD from *n* = 2 or 3 biologically independent samples. Statistical analyses were performed using one-way ANOVA followed by Dunnett’s multiple comparison test, **P < 0.01, ***P < 0.001

In addition to the core biosynthetic gene cluster, the biosynthesis of FR901379 involves a complex metabolic network. To investigate transcriptional changes in the chassis metabolic pathways of the high-producing strain, we performed Kyoto Encyclopedia of Genes and Genomes (KEGG) pathway enrichment analysis on transcriptome data of the overexpression strain OE*CehdaA*. The results revealed upregulation of genes associated with the biosynthesis of acetyl-CoA, palmitic acid, and 3′-phosphoadenosine-5′-phosphosulfate (PAPS), although genes involved in the fatty acid *β*-oxidation pathway were downregulated (Fig. 5A, B).

Within the acetyl-CoA biosynthetic pathway, transcription of pyruvate dehydrogenase complex (E1 and E2) was upregulated by 4.0- to 5.6-fold. Concurrently, transcription of aldehyde dehydrogenase (ALDH) and alcohol dehydrogenase (ADH) in the acetate to ethanol conversion pathway was significantly suppressed, thereby reducing acetyl-CoA consumption and ensuring its sufficient supply for palmitic acid (C16:0) synthesis. Furthermore, in the palmitic acid biosynthetic pathway, transcription of acetyl-CoA carboxylase (ACC1) and fatty acid synthase (FAS1 and FAS2) was increased by 4.6-fold, 5.6-fold, and 4.9-fold, respectively. In contrast, transcription of some key enzymes involved in *β*-oxidation of palmitic acid (including ACOX and ACAC) was downregulated. These changes suggest an enhanced flux of palmitic acid into the FR901379 biosynthetic pathway, ultimately leading to significantly increased FR901379 production in the overexpression strain OE*CehdaA*.

The sulfation of L-homotyrosine in the biosynthesis of FR901379 requires PAPS as the sulfate donor. The biosynthesis of PAPS involves a two-step process, the first step is catalyzed by ATP sulfurylase (ATPS) and the subsequent step is catalyzed by adenylylsulfate kinase (APSK) [38]. In the OE*CehdaA* strain, transcription of ATPS and APSK was significantly upregulated by 24.2-fold and 8.0-fold, respectively (Fig. 5B). However, metabolic flux from PAPS toward sulfide was also enhanced. Collectively, these results indicate that transcriptional upregulation of PAPS biosynthetic genes and the redirection of sulfur metabolic flux are critical for enhancing FR901379 biosynthesis.

### Functional validation of fatty acid synthesis pathway

To assess the contributions of fatty acid metabolism to FR901379 biosynthesis, we overexpressed *FAS1* and *ACC1* in MEFC10 strain. However, individual overexpression of either *FAS1* or *ACC1* did not increase FR901379 production (Fig. S5), suggesting that overexpression of single fatty acid biosynthetic genes alone is insufficient to raise palmitic acid levels and thereby failed to improve FR901379 production. We next supplemented the fermentation medium MKF with additional palm oil (a source of palmitic acid) at various concentrations during the culture of MEFC09. FR901379 production was increased under all tested conditions, with the highest improvement observed at 20 g/L palm oil. At this concentration, the FR901379 titer reached 666.0 mg/L, representing an 87.6% increase compared to the control (Fig. 5C). These results demonstrate that impaired transcription of palmitic acid biosynthetic genes negatively affects FR901379 production, whereas enhancing the supply of palmitic acid promotes its biosynthesis.

## Discussion

In filamentous fungi, the biosynthesis of secondary metabolites is strongly influenced by various untargeted regulatory mechanisms, including histone modifications and global regulators. The targets of these mechanisms often vary among different fungal strains. Rational modification of such untargeted regulatory factors can effectively alter the production of target metabolites. For example, Zhang et al. overexpressed the global regulator LaeA in *Monascus purpureus*, leading to enhanced production of monacolin K [21]. It is noteworthy that the same regulatory mechanism may produce divergent effects in different fungal species. In some filamentous fungi, HDAC acts as a negative regulator. Knockout of HDAC upregulates the biosynthetic pathway of sterigmatocystin, penicillin, and terrequinone A [20, 23, 24]. Treatment with HDAC inhibitors markedly enhances both the structural diversity and titer of secondary metabolites. Conversely, in some fungi, such as *Aspergillus fumigatus*, HDAC acts as a positive regulator, where knockout of *hdaA* was shown to reduce gliotoxin production [39]. Therefore, untargeted regulation acts as a key handle that is connected to numerous unknown metabolic modules and distinct physiological phenotypes. Based on this, we propose an URPS to uncover critical modules involved in the formation of important phenotypes such as high production.

This strategy involves perturbing the intracellular metabolic network by knocking out or overexpressing untargeted regulatory factors, thereby generating a library of mutant strains with diverse phenotypes. Subsequently, through analytical methods such as multi-omics analysis, correlations between specific phenotypes and metabolic modules are established, enabling the further proposal of formation mechanisms of important phenotypes. In general, generating a mutant library via mutagenesis breeding requires multiple rounds of screening, whereas URPS requires only a single gene knockout. In addition, mutagenesis commonly produces various mutation types, including SNPs (single nucleotide polymorphisms), InDels (insertions and deletions), SVs (structural variations), and CNVs (copy-number variations). Furthermore, different mutant strains can exhibit differences in their mutation types. By contrast, the metabolic perturbations generated by URPS are controllable and traceable, thereby ensuring the efficient and precise identification of relevant modules via omics analysis. In contrast to the rational engineering of synthetic pathways, which requires one-by-one validation, URPS allows for a more rapid inclusion of a broader range of candidate modules and targets into the analysis. Thus, URPS represents an effective method for investigating the mechanisms underlying critical phenotypes, particularly in microorganisms with complex metabolic networks and unclear genetic backgrounds, such as filamentous fungi.

Using the URPS approach, we found that the metabolic pathways involving acetyl-CoA, palmitic acid, and PAPS were key bottlenecks restricting FR901379 biosynthesis in the industrial strain *C. empetri* MEFC09. Transcriptomic analysis of the mutant strain revealed coordinated alterations in multiple precursor-supply pathways essential for FR901379 biosynthesis. In the high-yielding strain OE*CehdaA*, key genes involved in acetyl-CoA (E1 and E2), palmitic acid (ACC1, FAS1, and FAS2), and PAPS (ATPS and APSK) biosynthesis were upregulated, while those related to *β*-oxidation were downregulated. These findings highlight the crucial importance of palmitic acid and PAPS availability in FR901379 production and suggest that optimization of these precursor pools could substantially enhance production. This finding was further validated by a palm oil supplementation experiment, the addition of 20 g/L palm oil to the fermentation medium increased FR901379 production in wild-type strain MEFC09 by 87.6%. Overall, this study establishes a foundation for future metabolic engineering and fermentation optimization strategies aimed at increasing FR901379 production.

It should be noted that this study has certain limitations. At present, metabolic pathway analysis has been performed only on the overexpression strain OE*CehdaA*. Following this work, subsequent studies will involve transcriptome sequencing of other overexpression mutants, including OE*CelaeA*, OE*CeveA*, and OE*CecclA*, to elucidate their regulatory preferences toward certain metabolic pathways. Building on this foundation, global regulatory factors with complementary functions will be selected for combinatorial expression to further increase the titer of FR901379. In addition, we will employ the established URPS to elucidate the regulatory mechanisms of other potential global regulatory factors in the biosynthesis of FR901379.

## Conclusion

This study establishes URPS as an effective approach for dissecting complex metabolic networks. By genetically perturbing genes of histone modification and global regulators, we generated diverse phenotypic variants and identified key metabolic bottlenecks limiting FR901379 production in industrial strain of *C. empetri*. Notably, overexpression of *CehdaA* increased FR901379 production by 170%, reaching 884.5 mg/L, while transcriptome analysis revealed significant upregulation of key genes in the biosynthetic pathways of palmitic acid and PAPS. Exogenous supplementation with palm oil further enhanced the titer by 87.6%, confirming the decisive role of precursor supply. Our integrated approach, combining rational genetic perturbation with transcriptome analysis, provides a generalizable framework for elucidating high-titer mechanism in filamentous fungi.

## Supplementary Information

Supplementary material 1.

## Data Availability

No datasets were generated or analysed during the current study.

## Abbreviations

URPS: Untargeted regulatory perturbation strategy
IFIs: Invasive fungal infections
TCA: Tricarboxylic acid cycle
PPP: Pentose phosphate pathway
CSN: COP9 signalosome
PDA: Potato dextrose agar
HPLC: High performance liquid chromatography
FPKM: Fragments per kilobase per million reads
HDAC: Histone deacetylase
NHEJ: Non-homologous end joining
BGC: Biosynthetic gene cluster
KEGG: Kyoto Gncyclopedia of Genes and Genomes
ALDH: Aldehyde dehydrogenase
ADH: Alcohol dehydrogenase
ACC: Acetyl-CoA carboxylase
FAS: Fatty acid synthase
ACOX: Acyl-CoA Oxidase
ACAC: Acetyl-CoA acyltransferase
GABA: 4-Aminobutanoic acid
PAPS: 3'-Phosphoadenosine-5'-phosphosulfate
ATPS: ATP sulfurylase
APS: Adenosine 5'-phosphosulfate
APSK: Adenylylsulfate kinase
CysH: Phosphoadenosine phosphosulfate reductase
CysJ: Sulfite reductase
GAD: Glutamate decarboxylase
SDH: Sorbitol dehydrogenase
SNPs: Single nucleotide polymorphisms
InDels: Insertions and deletions
SVs: Structural variations
CNVs: Copy-number variations
