# Supplementary material for: Identifying metabolic bottlenecks for micafungin precursor production via untargeted regulatory perturbation
Source: Biotechnol Biofuels Bioprod. 2026 Jan 14;19:17. doi: 10.1186/s13068-026-02737-7 (PMC12874660; doi:10.1186/s13068-026-02737-7)
Supplement: Supplementary file 1 — Supplementary material 1. [file 13068_2026_2737_MOESM1_ESM.docx]

**Supporting Information**

**Identifying metabolic bottlenecks for micafungin precursor production via untargeted regulatory perturbation**

Ping Men ^1,2,3†^, Li Xie ^1,2,3†^, Jiachen Wang ^1,2,3,5^, Yu Zhou ^1,2,3,6^, Xiaoxi Zhang ^1,2,3^, Yanping Li ^7^, Xuenian Huang ^1,2,3,4^*, Xuefeng Lu ^1,2,3,4,8^*

^1^ Shandong Provincial Key Laboratory of Synthetic Biology, Qingdao Institute of Bioenergy and Bioprocess Technology, Chinese Academy of Sciences, Qingdao 266101, China.

^2^ Shandong Energy Institute, Qingdao 266101, China.

^3^ Qingdao New Energy Shandong Laboratory, Qingdao 266101, China.

^4^ University of Chinese Academy of Sciences, Beijing 100049, China.

^5^ College of Life Sciences, Qingdao Agricultural University, Qingdao 266109, China

^6^ Institute for Smart Materials & Engineering, University of Jinan, Jinan 250022, China

^7^ State Key Laboratory of Food Science and Resources, Nanchang University, Nanchang 330047, China

^8^ Marine Biology and Biotechnology Laboratory, Qingdao National Laboratory for Marine Science and Technology, Qingdao 266237, China.

^†^Ping Men and Li Xie contributed equally to this work.

*Correspondence to: Xuenian Huang ([huangxn@qibebt.ac.cn),](mailto:huangxn@qibebt.ac.cn),) Xuefeng Lu ([lvxf@qibebt.ac.cn](mailto:lvxf@qibebt.ac.cn))

**Table S1** Primers used in this study.

| **Primers** | **Sequence (5’→3’)** | **Notes** |
| --- | --- | --- |
| hph-F | ttcgggatcgcaagcgtaaag | Amplifying the cassette of *hph* |
| hph-R | caattatctttgcgaacccagg | Amplifying the cassette of *hph* |
| U-CecsnE-F | tcgcattggtatattgcatcga | Amplifying the upstream of *CecsnE* |
| U-CecsnE- hph -R | ctttacgcttgcgatcccgaaacgggcaaccgaaatgcgtccg | Amplifying the upstream of *CecsnE* |
| D-CecsnE-hph-F | cctgggttcgcaaagataattgaaaggtgtggataaagatctgga | Amplifying the downstream of *CecsnE* |
| D-CecsnE-R | ggtatctacaaaagaatcatgcagc | Amplifying the downstream of *CecsnE* |
| U-CecsnE-CS-F | ggggtttacagatgctaataactg | Amplifying the cassette of *CecsnE*-*hph* |
| D-CecsnE-CS-R | ctcgatgagactccagcaga | Amplifying the cassette of *CecsnE*-*hph* |
| U-CehdaA-F | cttaagatcacctgattgtggt | Amplifying the upstream of *CehdaA* |
| U-CehdaA-hph-R | ctttacgcttgcgatcccgaactgcatgatgacccggtgggcg | Amplifying the upstream of *CehdaA* |
| D-CehdaA-hph-F | ccctgggttcgcaaagataattgagagacaacccattaaatgcta | Amplifying the downstream of *CehdaA* |
| D-CehdaA-R | acaaacaagacgatctgcca | Amplifying the downstream of *CehdaA* |
| U-CehdaA-CS-F | ggctcttcataaagcttggagc | Amplifying the cassette of *CehdaA*-*hph* |
| D-CehdaA-CS-R | cgtcagagccatagcgtttc | Amplifying the cassette of *CehdaA*-*hph* |
| U-CecclA-F | gcatagatagcagaattcacctct | Amplifying the upstream of *CecclA* |
| U-CecclA-hph-R | ctttacgcttgcgatcccgaactggtagagtaaaattggcgg | Amplifying the upstream of *CecclA* |
| D-CecclA-hph-F | cctgggttcgcaaagataattgccgattgttacggttacggtc | Amplifying the downstream of *CecclA* |
| D-CecclA-R | actcacgcaatgcccttttg | Amplifying the downstream of *CecclA* |
| U-CecclA-CS-F | agttcatgagtttcgggaact | Amplifying the cassette of *CecclA*-*hph* |
| D-CecclA-CS-R | ctatcaatcagtcgcaactcc | Amplifying the cassette of *CecclA*-*hph* |
| U-CelaeA-F | gtctggaatcacacaagtcg | Amplifying the upstream of *CelaeA* |
| U-CelaeA-hph-R | tttacgcttgcgatcccgaaatcgattgcccatattcccg | Amplifying the upstream of *CelaeA* |
| D-CelaeA-hph-F | tgggttcgcaaagataattgccctcgcatacaatactgag | Amplifying the downstream of *CelaeA* |
| D-CelaeA-R | ttcttgcatactccctccaac | Amplifying the downstream of *CelaeA* |
| U-CelaeA-CS-F | tacatccagttgctttggcc | Amplifying the cassette of *CelaeA*-*hph* |
| D-CelaeA-CS-R | ggcaatggatggcatgttat | Amplifying the cassette of *CelaeA*-*hph* |
| U-CeveA-F | aagtgaaggccgtgtcatag | Amplifying the upstream of *CeveA* |
| U-CeveA-hph-R | tttacgcttgcgatcccgaattggctgagaaaacagtgaa | Amplifying the upstream of *CeveA* |
| D-CeveA-hph-F | tgggttcgcaaagataattgaccaagctcctcaataccat | Amplifying the downstream of *CeveA* |
| D-CeveA-R | catgtcgacacctccctaaa | Amplifying the downstream of *CeveA* |
| U-CeveA-CS-F | aagcgccaaacccaagaatc | Amplifying the cassette of *CeveA*-*hph* |
| D-CeveA-CS-R | gcgcaaatgtaggtgctaga | Amplifying the cassette of *CeveA*-*hph* |
| U-CevelB-F | gtagccgctttgggtaaagt | Amplifying the upstream of *CevelB* |
| U-CevelB-hph-R | tttacgcttgcgatcccgaatacgcaaggcggcggagtga | Amplifying the upstream of *CevelB* |
| D-CevelB-hph-F | tgggttcgcaaagataattgcaaagcgaatatatcccggc | Amplifying the downstream of *CevelB* |
| D-CevelB-R | ccctaccttgttttgacgtg | Amplifying the downstream of *CevelB* |
| U-CevelB-CS-F | tggttcactcgagagaacgt | Amplifying the cassette of *CevelB*-*hph* |
| D-CevelB-CS-R | cgcatccatttgcatagacg | Amplifying the cassette of *CevelB*-*hph* |
| PgpdAt-F | gttacactctgggaggatcc | Amplifying the cassette of P*gpdAt*- *CecclA*/*CehdaA*/*CelaeA*/*CeveA*/*FAS1*/*ACC1*-T*pgk* |
| Tpgk-R | attgcagcgcacaagtcagt |  |
| CehdaA-PgpdAt-F | acaactcatcaatcatcacatctagaatggagccgagcagcggcat | Amplifying the gene of *CehdaA* |
| CehdaA-Tpgk-R | acaaaattcttcatttatttactacctcgatgactcctcct | Amplifying the gene of *CehdaA* |
| CehdaA-CDS-F | aatggagggcgttgaatcag | PCR verification of strain OE*CehdaA* |
| CecclA-PgpdAt-F | acaactcatcaatcatcacatctagaatgccgatgtcaccagaacaa | Amplifying the gene of *CecclA* |
| CecclA-Tpgk-R | acaaaattcttcatttatttatcagaaatgtagagaagcga | Amplifying the gene of *CecclA* |
| CelaeA-PgpdAt-F | acaactcatcaatcatcacatctagaatgccgctcacagcccctcc | Amplifying the gene of *CelaeA* |
| CelaeA-Tpgk-R | caaaattcttcatttatttatcaagcctcggcaggtcggc | Amplifying the gene of *CelaeA* |
| CelaeA-CDS-F | ctatgtcgaatgtaggccct | PCR verification of strain OE*CelaeA* |
| CeveA-PgpdAt-F | acaactcatcaatcatcacatctagaatggcatccgccactgcgag | Amplifying the gene of *CeveA* |
| CeveA-Tpgk-R | caaaattcttcatttatttactactccagggcgggaagctc | Amplifying the gene of *CeveA* |
| CevelB-PgpdAt-F | acaactcatcaatcatcacatctagaatgcaagctcaggcgtatcc | Amplifying the gene of *CevelB* |
| CevelB-Tpgk-R | caaaattcttcatttatttatcaataagcatcatcttcttc | Amplifying the gene of *CevelB* |
| CevelB-CDS-F | gaccatcgcatttcgaacct | PCR verification of strain OE*CevelB* |
| M13-F | tgtaaaacgacggccagt | PCR verification of plasmids pU-CecclA/CehdaA/CelaeA/CeveA/FAS1/ACC1/cemelR |
| M13-R | caggaaacagctatgac |  |
| (pUC19)-UcemelR-F | gctatgaccatgattacgccaagcttgtttggcttatcccaccgaa | Amplifying the upstream of *cemelR* |
| UcemelR-(DcemelR)-R | tagaatccgtgcatagagattctagattcgaccgagttcgttcgct | Amplifying the upstream of *cemelR* |
| DcemelR-F | atctctatgcacggattctatagat | Amplifying the downstream of *cemelR* |
| DcemelR-(pUC19)-R | agctcggtacccggggatccctcgaggtagcatgtagcttacagcg | Amplifying the downstream of *cemelR* |
| FAS1-PgpdAt-F | acaactcatcaatcatcacatctagaatgacccccgaagtcgagca | Amplifying the gene of *FAS1* |
| FAS1-Tpgk-R | caaaattcttcatttatttactaaaacgtcgcaacagcaa | Amplifying the gene of *FAS1* |
| ACC1-PgpdAt-F | acaactcatcaatcatcacatctagaatgacggaagttgaggtaaa | Amplifying the gene of *ACC1* |
| ACC1-Tpgk-R | caaaattcttcatttatttactaatgcgaactcaaaaatt | Amplifying the gene of *ACC1* |
| (UcemelR)-PgpdAt-F | agcgaacgaactcggtcgaagttacactctgggaggatcc | Amplifying the cassette of P*gpdAt*-*FAS1*/*ACC1*-T*pgk* |
| Tpgk-(DcemelR)-R | tagaatccgtgcatagagatattgcagcgcacaagtcagt |  |
| UcemelR-CS-F | ttagttgctggagcttccgc | Amplifying the cassette of UcemelR- P*gpdAt*-*FAS1*/*ACC1*-T*pgk-*DcemelR |
| DcemelR-CS-R | cggaggatttcaaacaatgcc |  |

**Table S2** Plasmids used in this study.

| **Plasmids** | **Characteristics** | **Reference** |
| --- | --- | --- |
| pXH2-1 | Carrying gene of *hph*, Amp^r^ | [1] |
| pU19-ZX | Carrying cassette of P*gpdAt* and T*pgk*, Amp^r^ | [2] |
| pU-CecclA | Carrying cassette of PgpdAt-CecclA-Tpgk, Amp^r^ | This study |
| pU-CehdaA | Carrying cassette of PgpdAt-CehdaA-Tpgk, Amp^r^ | This study |
| pU-CelaeA | Carrying cassette of PgpdAt-CelaeA-Tpgk, Amp^r^ | This study |
| pU-CeveA | Carrying cassette of PgpdAt-CeveA-Tpgk, Amp^r^ | This study |
| pU-FAS1 | Carrying cassette of PgpdAt-FAS1-Tpgk, Amp^r^ | This study |
| pU-ACC1 | Carrying cassette of PgpdAt-ACC1-Tpgk, Amp^r^ | This study |
| pU19 | Empty carrier, Amp^r^ | This study |
| pU-cemelR | Carrying cassette of UcemelR-DcemelR, Amp^r^ | This study |
| pU-cemelR-FAS1 | Carrying cassette of UcemelR-PgpdAt-FAS1-Tpgk-DcemelR, Amp^r^ | This study |
| pU-cemelR-ACC1 | Carrying cassette of UcemelR-PgpdAt- ACC1-Tpgk-DcemelR, Amp^r^ | This study |


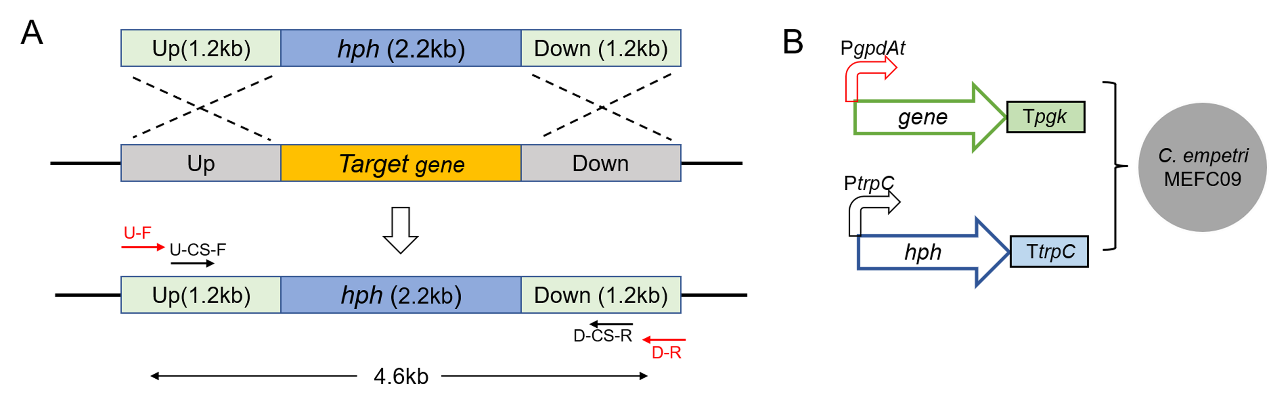


**Fig. S1** Schematic diagrams of the expression element. **A** The expression element for gene disruption in MEFC10; **B** The expression element for gene overexpression in *C. empetr* MEFC09


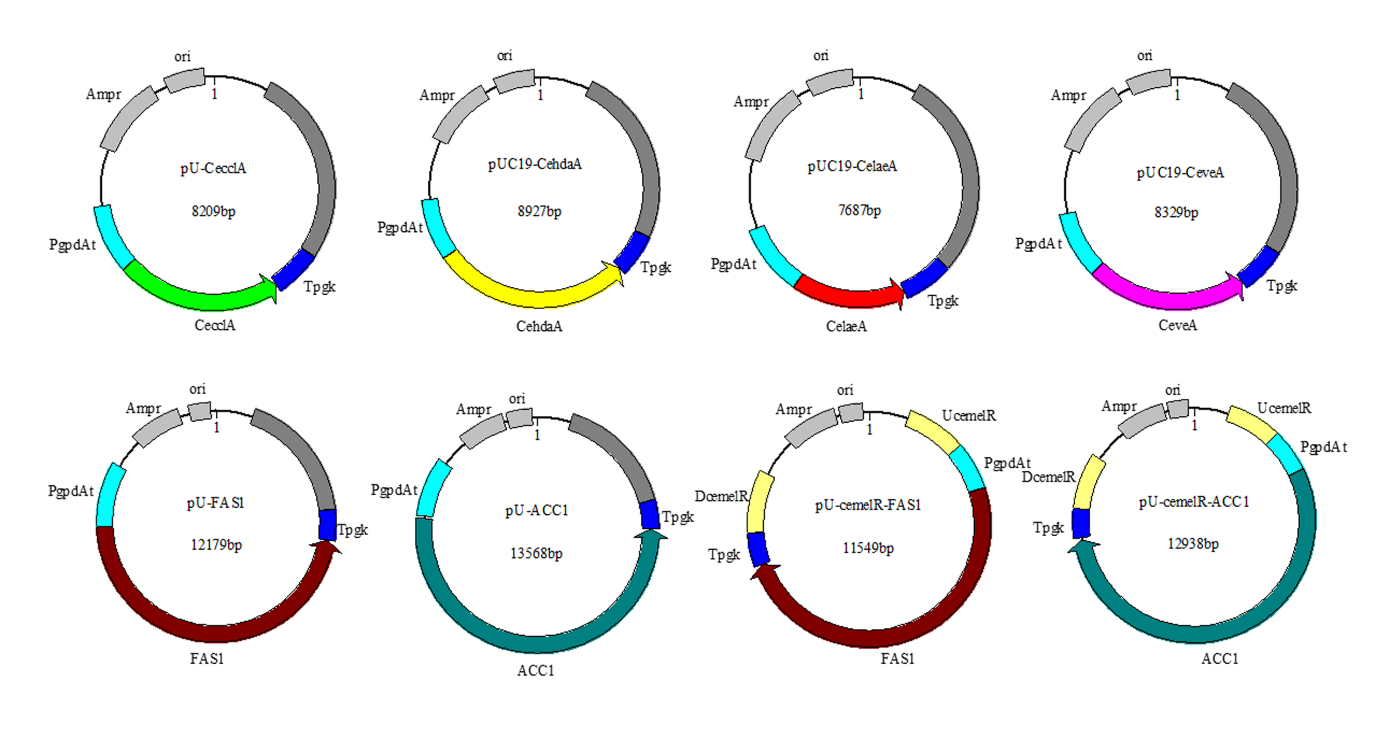


**Fig. S2** The recombinant plasmids for gene overexpression


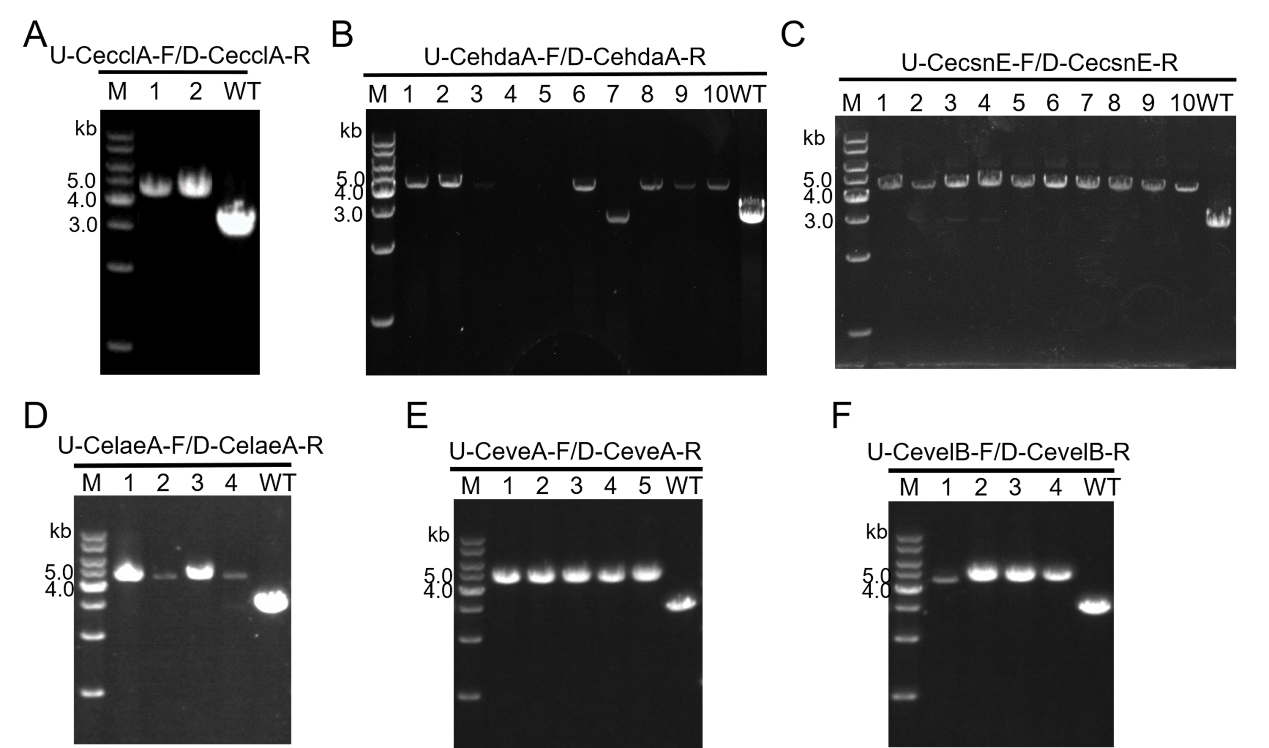


**Fig. S3** The genomic PCR verification results of deletion transformants. **A**: ∆*CecclA*; **B**: ∆*CehdaA*; **C**: ∆*CecsnE*; **D**: ∆*CelaeA*; **E**: ∆*CeveA*; **F**: ∆*CevelB*; Lane M: 1 kb DNA marker; lane 1-10: transformants; lane WT: MEFC10


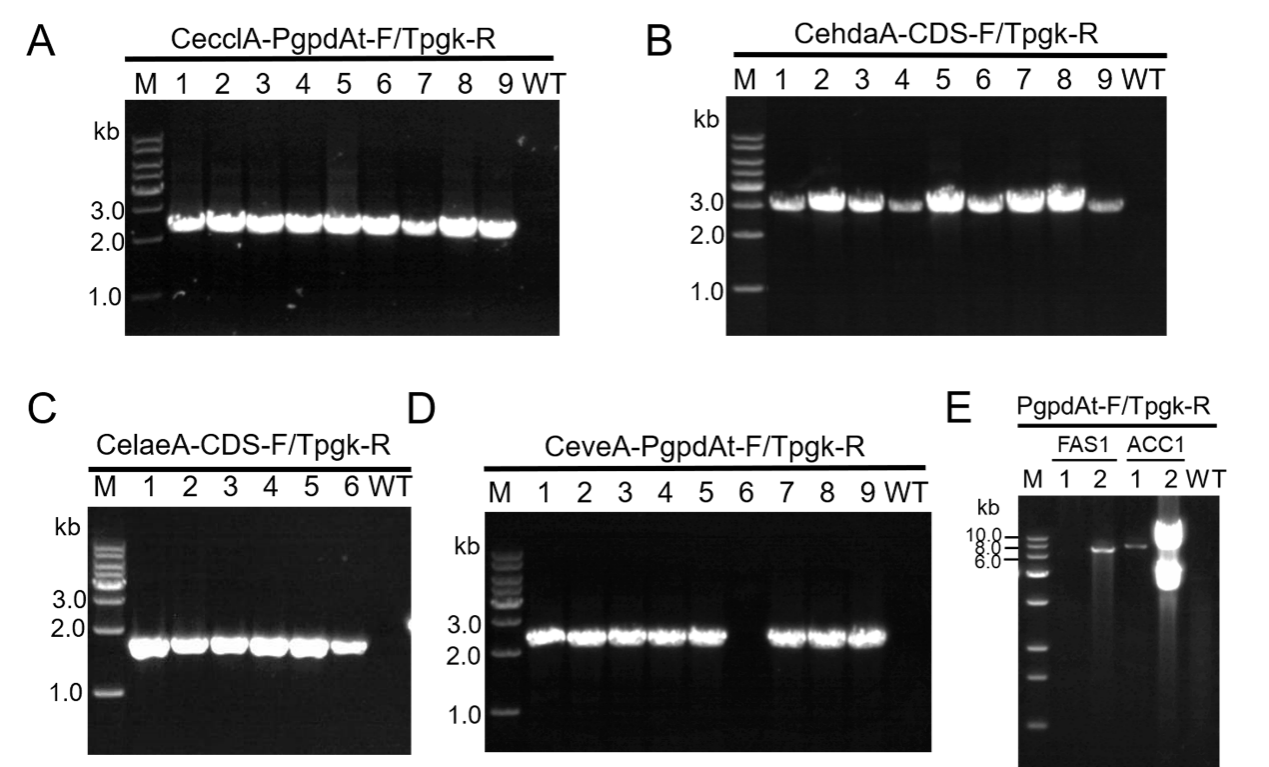


**Fig. S4** The genomic PCR verification results of genes overexpression transformants. **A:** OE*CelaeA*; **B:** OE*CeveA*; **C:** OE*CecclA*; **D:** OE*CehdaA*; **E:** OE*FAS1* and OE*ACC1.* Lane M: 1 kb DNA marker; lane 1-9: transformants; lane WT: MEFC09


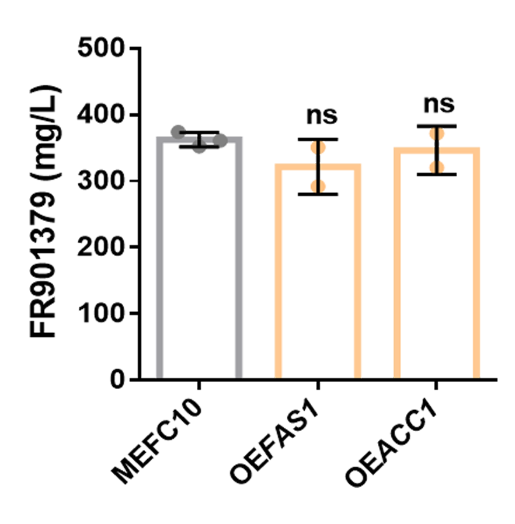


**Fig. S5** Titers of FR901379 in strains MEFC10, OE*FAS1* and OE*ACC1*. All data are mean ± SD from n = 2 or 3 biologically independent samples. Statistical analyses were performed using one-way ANOVA followed by Dunnett’s multiple comparison test. ns: no significant difference

**References**

[1] Huang X, Lu X and Li JJ. Cloning, characterization and application of a glyceraldehyde-3-phosphate dehydrogenase promoter from *Aspergillus terreus*. J Ind Microbiol Biotechnol. 2014;41(3): 585-92. <https://doi.org/10.1007/s10295-013-1385-0>.

[2] Men P, Zhou Y, Xie L, Zhang X, Zhang W, Huang X, et al. Improving the production of the micafungin precursor FR901379 in an industrial production strain. Microb Cell Fact. 2023;22(1): 44. <https://doi.org/10.1186/s12934-023-02050-0>.
